# A review of parameters and heuristics for guiding metabolic pathfinding

**DOI:** 10.1186/s13321-017-0239-6

**Published:** 2017-09-15

**Authors:** Sarah M. Kim, Matthew I. Peña, Mark Moll, George N. Bennett, Lydia E. Kavraki

**Affiliations:** 1 Department of Computer Science, Rice University, 6100 Main St., Houston, TX 77005 USA; 2 Department of BioSciences, Rice University, 6100 Main St., Houston, TX 77005 USA

**Keywords:** Metabolic pathfinding, Graph-based search, Metabolic engineering

## Abstract

Recent developments in metabolic engineering have led to the successful biosynthesis of valuable products, such as the precursor of the antimalarial compound, artemisinin, and opioid precursor, thebaine. Synthesizing these traditionally plant-derived compounds in genetically modified yeast cells introduces the possibility of significantly reducing the total time and resources required for their production, and in turn, allows these valuable compounds to become cheaper and more readily available. Most biosynthesis pathways used in metabolic engineering applications have been discovered manually, requiring a tedious search of existing literature and metabolic databases. However, the recent rapid development of available metabolic information has enabled the development of automated approaches for identifying novel pathways. Computer-assisted pathfinding has the potential to save biochemists time in the initial discovery steps of metabolic engineering. In this paper, we review the parameters and heuristics used to guide the search in recent pathfinding algorithms. These parameters and heuristics capture information on the metabolic network structure, compound structures, reaction features, and organism-specificity of pathways. No one metabolic pathfinding algorithm or search parameter stands out as the best to use broadly for solving the pathfinding problem, as each method and parameter has its own strengths and shortcomings. As assisted pathfinding approaches continue to become more sophisticated, the development of better methods for visualizing pathway results and integrating these results into existing metabolic engineering practices is also important for encouraging wider use of these pathfinding methods.

## Background

Metabolic engineering is the scientific process of manipulating the metabolism of a microorganism to produce valuable compounds. Engineering microbial production involves the disruption of endogenous genes or adding genes from heterologous organisms to form pathways that tap into the natural metabolic network. There have been numerous successes of metabolic engineering, including the well publicized biosynthesis of artemisinic acid, a precursor to the antimalarial drug artemisinin [1], and thebaine, a precursor to hydrocodone and morphine [2]. In each of these cases, a pathway responsible for the production in plants was translated to a chassis microorganism, such as *E. coli* and *S. cerevisiae*, to separate the supply of these therapeutics from the plants they were sourced from. At the root of these successes is the identification of the requisite pathways and the systematic transfer of these pathways to a microbial host.

Metabolic pathfinding has clear applications to the first step in the design-build-test-learn cycle for developing biosynthetic pathways [3]. We define metabolic pathfinding as the process of identifying viable routes through a metabolic network from a starting compound to a desired target compound. Here, pathways are not limited to those that exist within a single organism, but can contain any enzymatic reactions from multiple organisms to complete a novel, heterologous pathway. To perform pathfinding we need a metabolic network that is constructed using information linking reactants to products through characterized enzymatic reactions. Several metabolic databases provide the requisite connectivity data used to construct a metabolic network structure. Of these, the Kyoto Encyclopedia of Genes and Genomes (KEGG) has been employed most frequently, likely due to being one of the first metabolic databases available with open access and a wide breadth of information. MetaCyc [4] also has descriptive entries for metabolic pathways that are attributed to many groups of organisms. Some databases, including BRENDA [5] and ExPASy [6], have more information about the enzymes including kinetics and protein structure, whereas others, such as ChEBI [7], specialize in descriptions of small molecules. New content is being continuously added to all these databases, many of which now source enzymatic reactions from thousands of organisms.

**Fig. 1:**
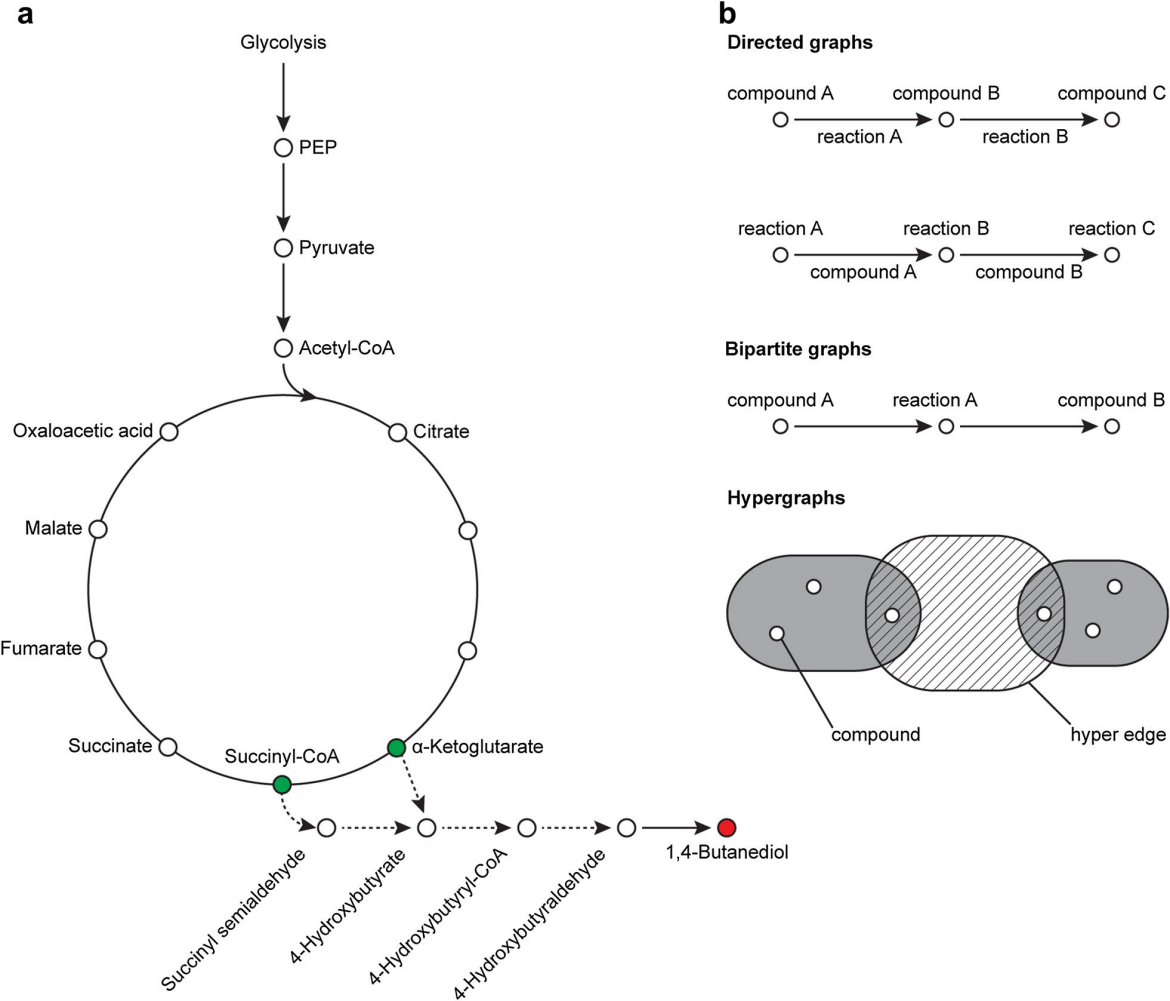
**a** 1,4-Butanediol is an example of a non-natural compound that is produced using a heterologous pathway generated by an pathway-identification algorithm. These heterologous genes from organisms differing from the host are dashed. Intermediates of the citric acid cycle were used as starting compounds in a pathway search restricted by path length toward the desired target, 1,4-butanediol. **b** Graph-based networks can be described as either directed, bipartite, or hypergraphs. In directed graphs, nodes (open circles) and edges (arrows) can represent either compounds or reactions. In the case of bipartite graphs, the pathway will be composed of nodes that alternate between compounds and reactions. For hypergraphs, groups of multiple compounds can be linked through a single hyperedge that may represent a reaction rule that links compounds that are associated with multiple additional reaction rules

Traditionally, researchers have manually searched existing literature and databases to design pathways. However, the rapidly growing body of metabolic information makes it difficult to effectively survey and utilize all available resources. Computational approaches have been developed to enable researchers to take advantage of these growing resources. For example, pathways for production of 1,4-butanediol, a non-natural compound, were discovered with the assistance of a pathway-identification algorithm [8]. Thousands of pathways, four to six reactions long, were generated starting from common central metabolites. Solution prioritization was required to whittle the pathways down to a manageable number to be constructed and tested in the lab resulting in a demonstration of feasibility for a novel, biocatalytic route (Fig. 1a). Assisted metabolic pathfinding may aid in the more rapid discovery of synthesis pathways for other valuable products.

Assisted metabolic pathfinding aims to solve two main challenges—the challenge of efficiently speeding up the pathway search process and the challenge of selectively finding biologically feasible, novel pathways. This paper focuses primarily on the approaches of pathfinding algorithms that address these two challenges. However, improvements in the search algorithms alone are not sufficient to solve these challenges, as the quality of the pathway results is also heavily dependent on the metabolic resources utilized by the search algorithm. Advancements in metabolic pathfinding rely on advancements in techniques for expanding the metabolic search space. For example, retrosynthesis-based approaches [9, 10] can be used to build search spaces that extend beyond the data stored in curated metabolic databases. Other databases like ATLAS [11] and XTMS [12] store information on extended search spaces and even apply existing pathfinding techniques (BNICE [13, 14] and RetroPath [15], respectively) to these spaces. Metabolic pathfinding may not be the main focus of retrosynthesis algorithms and expanded databases; however, these resources are nevertheless critical for finding novel metabolic pathways and will be included in this review.

The metabolic pathfinding problem itself can be further divided into two different approaches: graph-based pathfinding and constraint-based pathfinding. This review will focus on graph-based pathfinding, which highlights the connections between compounds and reactions in the metabolic network. Graph-based approaches represent a metabolic pathway as a path that consists of an ordered series of intermediate compounds and reactions that transform some defined starting compound(s) to some defined target compound(s). Graph-based pathfinding utilizes a very well-studied data structure to represent the metabolic network, abstracting away more complicated interactions between compounds and enzymes in the cell. This abstraction enables graph-based methods to readily scale with larger metabolic networks spanning multiple organisms. However, since much of the underlying metabolic network is abstracted by the graph representation, there is a greater chance for graph-based approaches to return pathways without biological significance unless relevant parameters and heuristics are introduced to guide the search. Constraint-based methods (e.g., [16]) highlight the stoichiometry and relative rates of reactions involved in the metabolic process being studied. In many constraint-based methods, a selected set of reactions is optimized to meet a specified objective (e.g., maximizing the yield of a valuable compound) under the steady state assumption, meaning that there is no net increase or decrease of metabolites within the studied system. For constraint-based methods, elementary flux modes or extreme pathways can serve as the representation of a metabolic pathway [17–19]. Unlike graph-based paths which may only include the main compounds and reactions in a pathway, elementary flux modes and extreme pathways provides a more complete summary of the requisite intermediate compounds and enzymes while conforming to steady-state constraints. Overall, constraint-based methods tend to offer a more accurate model of a known metabolic network, such as one from a well-studied organism like *E. coli*. However, this approach is not yet able to computationally scale to very large metabolic networks [20]. Though algorithms have been developed to identify viable pathways using elementary mode analysis [20, 21], we choose to focus specifically on graph-based pathfinding to examine how parameters and heuristics can be used to efficiently guide the search in large-scale metabolic networks.

A metabolic network can be described as connections between compounds and the enzymes catalyzing reactions between compounds, which lends itself well to graph representation. There are many different ways a metabolic network can be represented as a graph (Fig. 1b). One of the simplest ways is for the nodes in a graph to represent the compounds in the metabolic network, and the edges to represent the reactions or enzymes that connect one compound to another. This representation is used in several earlier pathfinding algorithms [22–24]. It is also possible for the nodes in a metabolic graph to represent the enzymatic reactions and the edges to represent the intermediate compounds, as done in MetaRoute [25]. Another possible graph representation of the metabolic network is for both compounds and reactions to be represented as nodes in a bipartite graph, where edges represent the connections between compounds and reactions. This representation is used in a few algorithms [26, 27]. A third possible graph representation is the hypergraph, where multiple compounds (i.e., the reactants) can be connected to multiple target compounds (i.e., the products) with a single hyperedge (the reaction). Unlike other graph representations, the hypergraph representation can connect two different groups of compounds with a single reaction hyperedge, which allows more details about each reaction (i.e., all intermediate compounds involved) to be shown explicitly in the representation [20]. The hypergraph representation is used in several pathfinding and retrosynthesis algorithms [12, 20, 28–30]. Node and edge weights based on relevant parameters (e.g., atom mappings, compound similarity, reaction thermodynamics, and organism-specific information) can be introduced to any of the above graph representations to guide the pathfinding search towards more biologically relevant results.

This review covers the techniques supporting graph-based metabolic pathfinding algorithms and the heuristics that guide pathway discovery from networks, enzymatic reactions, and chemical structures to a specific host organism context (Fig. 1b). We will begin with a description of the structure of the metabolic network in terms of (1) graph connectivity, which refers to the number of connections each node has across the network, and (2) path length, or the number of transformative steps that separate any two compounds in the network (“Metabolic network structure” section). Then, the role of compound structure (“Structure of compounds” section) and reaction specific information (“Reactions” section) in identifying feasible, novel pathways will be discussed. Next, we briefly describe the role of organism-related information (“Organism” section). We conclude the paper with a discussion of the limitations and implications for future directions for metabolic pathfinding (“Discussion” section). By describing the advantages and disadvantages of features used in current pathfinding approaches, we hope to guide interested users to the algorithms that suit their needs while summarizing the latest research for developers.

## Metabolic network structure

Properties of the metabolic network representation can be used to guide and constrain the search problem and rank the resulting pathways. The properties that have been used in the literature are the connectivity of the network and the length of pathways found. The individual compounds and reactions of a pathway can also be assigned weights based on biochemical and network-based properties.

### Graph connectivity

**Fig. 2:**
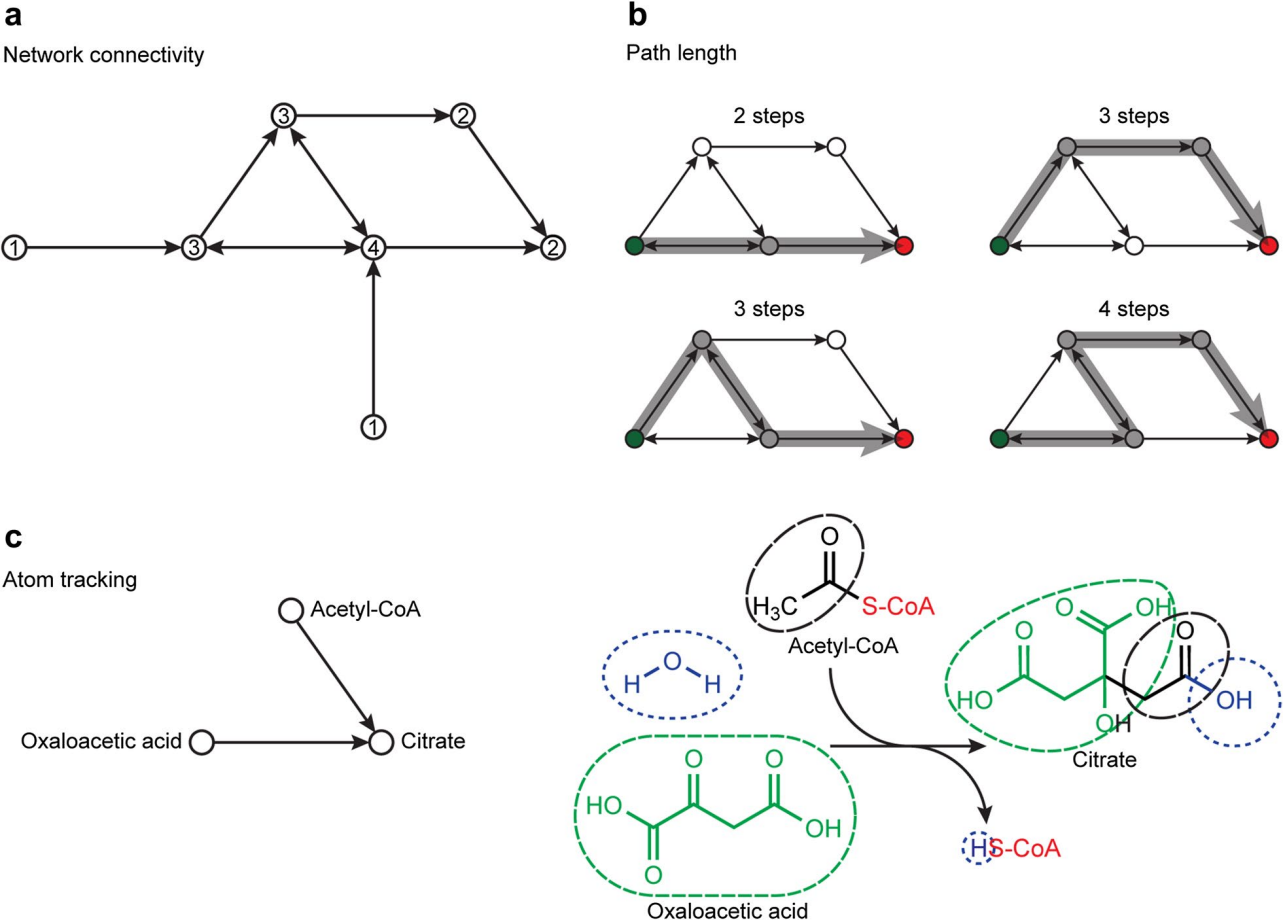
**a** The degree of connectivity for a node (vertex) within a metabolic network can be described by the number edges that are incident to it. In this case, the degree for each node, or the total number of incoming and outgoing edges in the directed graph, is provided in each node. **b** The path length can be easily determined by counting the number of edges that must be traversed to complete a pathway from the starting (green) node to the target (red) node. This is directly analogous to the number of enzymatic steps that must be engineered to complete a biosynthetic pathway. **c** Atom mapping is a highly detailed interpretation of chemical transformation that occurs along each edge of the metabolic network. Here, the first step of the citric acid cycle can be both described as a simple network with two edges and three nodes, or as a balanced chemical reaction where atoms can be tracked between reactants and their corresponding products. Notice that the network ignores water and coenzyme A (CoA), whereas the atom mapped reaction can be used to identify what atoms from the reactants acetyl-CoA and oxaloacetic acid contributed to the product, citrate

The graph-based representation of the network makes it intuitive to gravitate towards graph-based features and constraints, particularly graph connectivity (Fig. 2a). Many approaches identify highly connected compound nodes in the graph, or hub compounds, which appear in many different reactions. Identifying hub compounds can suggest potential currency metabolites, or side compounds that are used as energy or electron providers but are not incorporated into the final product compounds (e.g., NADH, ATP, etc.). As such, pathways routing through currency metabolites tend to not be biologically meaningful, and for many algorithms, these currency compounds are manually removed [13, 14, 24, 31, 32]. In Croes et al. [31], the weight of compound vertices is set equal to the degree of the compound in the network, biasing the search against going through highly connected compounds. Croes et al. compared this weighted graph search with an unweighted graph and a filtered graph (where 36 highly connected pool metabolites were removed), and found that weighted graph search performed better (85% correspondence with annotated pathways) than the unweighted graph search (30%) and filtered graph search (65%). Croes et al. also suggested that the small world property of metabolic networks described by Wagner and Fell [33] is an artifact of having currency metabolites in unweighted metabolic graphs, which make compounds in the metabolic network seem more tightly connected. This is also suggested by several other papers [34–36].

In Faust et al. [26], different weighting schemes for compounds and reactant pairs (RPAIRs) were compared amongst each other. The weighting schemes included weighting compounds by degree, as described by Croes et al. in 2006, and weighting RPAIRs by their classification type. The RPAIR classification can be treated as a ranking for how relevant the pair of compounds are in the reaction. For example, if an RPAIR is classified as “main,” the compounds involved in the RPAIR are considered the main chemical transformation that occurs in the reaction, whereas a RPAIR classified as “cofac” or “ligase” may describe compounds that serve as metabolite compounds or facilitators of the reaction. Faust et al. introduces higher weights for RPAIR classifications that are considered less relevant to the reaction, favoring pathways that include more RPAIRs classified as “main.” According to this study, searches using the Croes et al. weighting for compounds found better results than searches without compound weighting, while using RPAIR classification weighting showed no significant improvement in search results.

In MetaRoute [25], the weight of the compound vertices is set to the sum of the out-degree of the compound and the context weight of the in-going reaction nodes. The context weight is based on the degree of the side compounds involved in the reaction. The context weighting gives rare compounds a high weight and common compounds a low weight, encouraging paths to go through reactions that use common compounds as side compounds.

The connectivity of a graph is very simple to compute, and it is no surprise that it has been used by several metabolic pathfinding approaches. Despite its simplicity, connectivity can be used to effectively infer some biochemical information about the metabolic network. However, excluding features of the metabolic network based on connectivity alone may not reflect known biochemical properties. For example, excluding highly connected compounds to avoid currency compounds may also exclude compounds that play a significant role in pathways (e.g., pyruvate). Unlike other algorithms, M-path by Araki et al. [37] uses hub compounds as a launch point to speed up the search. The approach identifies 139 compounds involved in eight or more reactions as hub compounds and introduces the reactions between the start compound and the hub compounds as the first steps in the search. Araki et al. refers to a paper by Barabasi and Oltvai [38], which suggests that highly connected compounds that are not currency metabolites are critical in linking together many compounds in the metabolic network. By including these highly connected compounds as first intermediates, the M-path algorithm can shorten the number of reaction steps needed to reach the target compound and improve the performance of the search.

### Path length

Pathfinding algorithms often optimize for pathways with the smallest number of enzymatic steps, as these pathways tend to require less manipulation in a metabolic engineering context (Fig. 2b). Many pathfinding algorithms set a maximum path length [31] or give the user an option to specify a maximum path length [23, 39, 40]. Pitkanen et al. [41] uses path length as part of the pathfinding heuristic to limit the search in the underlying networks. Pathways can also be ranked based on path length (e.g., algorithms finding *k*-shortest paths [27, 42]). Ranking by path length is often a byproduct of the applied graph search algorithm (i.e., *k*-shortest paths) and used to organize pathway results. In order to distinguish pathfinding methods that actively include path length as a constraint or heuristic from methods that only use path length to rank pathway results, the latter cases were not marked as using path length in Table 1. Since path length is an inherent property of the solution pathway, no additional computation is required for obtaining this information. However, strongly biasing the search towards shorter pathway results ignores longer pathway results that may be equally valid for lab testing, such as the pathway for synthesizing a precursor of opioids that took 23 enzymatic steps in lab [2].

**Table 1:**
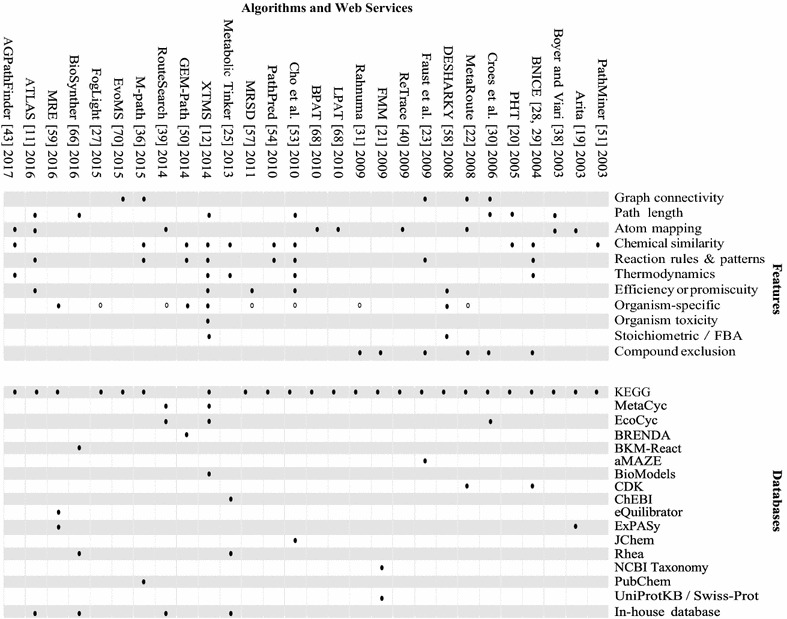
Algorithms are arranged by publication date, and closed circles denote the features used in the respective pathfinding algorithms along with the databases they draw information from

Open circles used in the organism-specific column indicate that users may input weights or parameters to make the algorithm organism-specific, but the algorithm itself does not provide options for the user to select for specific organisms

## Structure of compounds

Most network representations include both structures of compounds and reactions, along with parameters that give additional information on both these parts. The chemical structure of compounds in the metabolic network can be useful in inferring the existence of a biochemical reaction between compounds, as biochemical reactions tend to have products that structurally resemble one or more reactants. Structural information can be represented at different levels of detail, which introduces a trade-off between the accuracy of the similarity measure and the computational complexity of the overall metabolic pathfinding problem.

### Atom tracking

At the finest level of detail, algorithms can track changes on atomic level (Fig. 2c). Retaining as many of the atoms from the start compound in the target compound automatically excludes currency metabolites that contribute no atoms to the final product, which helps exclude pathway results that are biochemically infeasible. Also, conserving as much of the atomic structure of compounds in each reaction step can help to select pathways that are more biologically feasible. This method was first introduced by Arita [22], which aims to conserve at least one atom from start compound to target using *k*-shortest paths. The MetaRoute algorithm [25] also uses this approach. Building on this approach, new algorithms aimed to conserve multiple atoms. Pitkanen et al. [41] uses a heuristic to maximize the number of carbons transferred during a reaction, while also minimizing the path length. This encourages the inclusion of reactions that transfer more carbon atoms in the final branched pathway results. In Heath et al. [27], the pathway must conserve a minimum number of carbon atoms from start to target compound. A search to find the maximum number of conserved carbon atoms will start with the total number of carbon atoms in either the start or target compound and then decrement this number by one if no pathways are found that conserve that number of atoms. In Boyer and Viari [39], pathways must conserve a minimum number of atoms which do not necessarily need to be carbons. In the initial carbon flux path algorithm proposed by Pey et al. [42], any reactions not involving a carbon exchange between its main reactant and product were removed from the search space. Pey et al. later updated their carbon flux paths algorithm to include atom tracking [43] to insure carbons from the start compound were eventually incorporated into the target compound. RouteSearch [40] maximizes atoms conserved throughout the pathway using a heuristic scoring function. This score accounts for five different atom types (carbon, oxygen, nitrogen, phosphorus, and sulfur), and each type of atom can be assigned a different weight. More recently, atom group tracking has been introduced by AGPathFinder [44]. Instead of tracking single atoms, this algorithm tracks groups of adjacent atoms connected by bonds. This avoids the computational cost of tracking individual atoms, but still captures much of the information gained by atom tracking. Incorporating atomic level information into the search ensures that at least a portion of the starting compound is used to produce the target compound, which may filter out many biologically infeasible pathways. In previous years, atom mapping information was not as readily available; however, as new methods have been developed to computationally predict atom mapping, more and more pathfinding algorithms have included atom tracking in the search. Tracking individual atoms can be computationally expensive, especially if every possible combination of atoms conserved from compound to compound is considered [45]. Even so, the fact that many recent pathfinding approaches incorporate atom tracking suggests it is an important parameter for the pathfinding problem.

### Chemical similarity

If two compounds have similar chemical structures, there is a decent chance that these compounds can be connected by a common reaction. Several approaches have used different representations of chemical structure as a way of guiding, constraining, and ranking the search.

#### Chemical fingerprint

Several approaches use chemical fingerprints and Tanimoto coefficients [46] to measure compound similarity. A chemical fingerprint is a binary vector consisting of a string of ones and zeros. Each bit represents whether the compound contains a certain structural feature, such as the number of single carbon, carbon bonds present in the compound and the presence of chemical functional groups or ring structures. There are many available compound fingerprints that include different numbers and types of structural features. The Tanimoto coefficient is used to measure the similarity between two different compounds and is calculated by dividing the total number of structural features shared between the two compounds by the total number of structural features contained in both compounds. In Pathway Hunter Tool (PHT) [23], chemical fingerprints are included in the metabolite mapping scoring function, which is calculated by summing the calculated chemical similarity score and percentage atomic mass contribution. The algorithm uses this score to determine which reactants and products will be connected by edges in their search graph.

#### Graph-based comparison

**Fig. 3:**
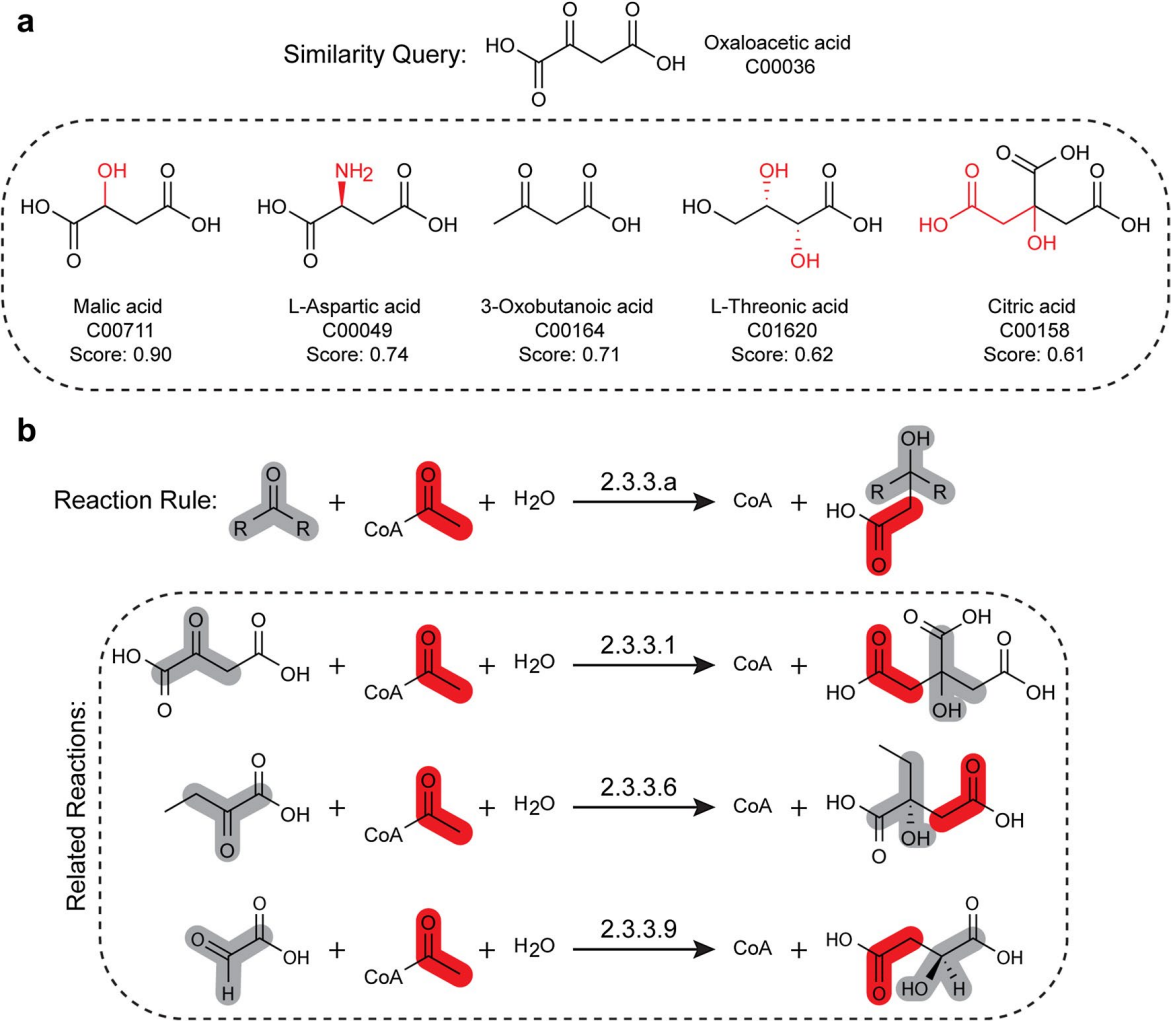
**a** Chemical similarity can be quantified by comparing common structural features. For example, SIMCOMP is an algorithm that calculates a similarity score by determining the maximum common substructure between two compounds. SIMCOMP can be used to rank all compounds in the KEGG database against a single queried compound, in this case oxaloacetic acid, by similarity score. The structural dissimilarities (substitutions and additions marked in red) of five familiar compounds relative to oxaloacetic acid are highlighted to provide context to the calculated similarity score. **b** Generalized reaction rules describe enzymatic reactions where the reactants share structural motifs and undergo related transformations within an EC class. Reaction rule 2.3.3.a is one of 86 BNICE generalized reaction rules described by Henry et al. [70]. Examples of enzymatic reactions described by this reaction rule include citrate (Si)-synthase (2.3.3.1) that forms citrate from oxaloacetate, 2-ethylmalate synthase (2.3.3.6) that forms 2-ethylmalate from 2-oxobutyrate, and malate synthase (2.3.3.9) that forms malate from glyoxylate. Motifs of the constituent reactants are highlighted in grey (aldehyde or ketone group) and red (acetyl group), and the corresponding atoms are identified in the products. Databases can be expanded, as is the case with MINEs and ATLAS, by applying reaction rules to metabolites that share a common motif

Other approaches rely on the graph representation of chemical compounds. Metabolic Tinker [28] uses a heuristic based on similarity of functional groups of atoms and bonds between the current compound and the target compound identified using a graph comparison technique similar to the one described in [47]. In this technique, each compound is represented as a graph, where atoms are the nodes and bonds are the edges. Common structural features between compounds are then identified by finding the maximal common subgraph(s). SIMCOMP [48], an algorithm that identifies the maximum common substructure between the graph representations of two compounds, was used for building the KEGG RPAIR database utilized by many pathfinding algorithms (Fig. 3a). SIMCOMP uses a variant of the Bron-Kerbosch maximum clique algorithm [49] to identify the maximum common substructure of two compounds.

Unlike chemical fingerprints, where a pre-determined set of chemical characteristics are used to compare two compounds, the graph comparison approach directly compares the chemical structure of two compounds against each other. The graph comparison approach tends to be more accurate in calculating structural similarity but is more computationally expensive [50]. In GEM-Path [51], both chemical fingerprints with Tanimoto coefficients and the subgraph matching of chemical structure are used to measure chemical similarity.

Calculating compound similarity is not as computationally expensive as atom mapping and serves as a check that the reactions included in pathways are biochemically feasible. However, compound similarity falls short in the cases where two compounds share many common structural components but are not biochemically related.

## Reactions

In addition to information about the compounds involved in the network, graph-based searches also include information on reactions. This information can be used to both constrain and expand the search to find novel pathways.

### Reaction rules

Building off the idea of structural similarity, some algorithms introduced reaction rules, or more general transitions between compounds based on changes in chemical structure. Two enzymatic reactions may involve different reactants and products; however, if the same structural change occurs between reactants and products in these reactions (i.e., functional group A is replaced by functional group B), these reactions may both fall under the same reaction rule. Reaction rules allow new, potentially feasible pathways to be found by introducing reactions that may not yet have been added to metabolic databases. These rules can both be used to (1) create a metabolic network without directly requiring information on enzymatic reactions from metabolic databases and (2) help expand an existing metabolic network created based on a metabolic database.

Reaction rules are based heavily on structural representations of compounds. In BNICE [13, 14], compounds are represented as an atom-bond matrix, and the reactions are represented as the difference between the matrices of the substrate and product compounds (Fig. 3b). With this more generalized representation of reactions, BNICE reduces the existing database of 43,000 enzymes to 250 generalized enzymatic reactions by grouping together enzymes that catalyze reactions which follow the same reaction rules. In PathMiner [52], each compound is similarly described as a set of 145 chemical descriptors (based on atoms/bond information), and reactions are represented as vector differences. The reactions are used as a heuristic to guide an A* search [53]. In M-path [37], compounds are represented by chemical feature vectors that account for 318 atom and bond feature types. Atom types include primary, secondary, and tertiary carbons, and each covalent bond in a compound is counted as a pair of atom types. Reactions are again represented as reaction feature vectors that describe difference in number of atom/bond feature types between substrates and products. In Cho et al. [54], there is a reaction rules database containing constructed reaction rules. PathPred [55] uses so-called RDM patterns from RPAIRs, which take into account the reaction center, the difference regions, and the matched regions between the reactants and products. PathPred also uses Jaccard coefficient [56] to compare compounds, and it weights the atoms closer to the reaction center more greatly compared to more distant atoms. A reaction score is calculated based on the Jaccard coefficient for each reaction, and the overall pathway score is the average of the reaction scores of all its reactions. In Faust et al. [26], RPAIR mappings are used without atom tracking to show the connectivity of compounds without annotations of atoms. In FMM [24], reactions are represented as a $16,\!884\times 16,\!884$ matrix, where each row and column represents a compound and having a ‘1’ represents that there exists a forward reaction between the compounds. In RetroPath by Carbonell et al. [15], the molecular signature of any given compound is defined by a subset of neighboring atoms and chemical bonds surrounding each individual atom in the compound. The reaction rules are defined as the differences in molecular signatures between the reactant compounds and product compounds in a reaction. Only the atoms and bonds within a given number of bonds away from each atom are considered as part of the molecular signature. This distance, referred to as the diameter by Carbonell et al., could be increased to include more surrounding atoms and bonds in the molecular signature and in turn, make each reaction rule include more detailed differences in molecular structure between reactants and products. Or, the diameter could be decreased to include less of the surrounding atoms and bonds in the molecular signature, causing each reaction rule to be more general and applicable to more groups of compounds. Thus, by changing the diameter, the strictness of reaction rules can be adjusted to prevent an exponential explosion of potential reactions. Reaction rules allow the search to find novel pathways not present in existing metabolic databases. However, the issue with using reaction rules to find new paths is that there is a potential for an exponential explosion of results.

### Thermodynamics

Another common feature taken into account by pathfinding algorithms is thermodynamic feasibility of the reactions in pathways. Almost all algorithms that include thermodynamics use the component contribution method [57] for calculating $\Delta$G. In MetabolicTinker [28], missing directional information is inferred from $\Delta$G. If it is not possible to calculate the $\Delta$G, the edge is treated as a bidirectional edge. The search heuristic is based partially on thermodynamics, and paths are ranked based on thermodynamic feasibility. In BNICE [14], the $\Delta$G value is used to analyze enzymatic reactions in different groups (profiling) and suggest feasibility of reactions. In Cho et al. [54], enzymes are ranked based on thermodynamic favorability, among other factors (such as binding site covalence and chemical similarity). The XTMS webserver [12] uses a scoring function to rank pathway results found by the RetroPath search algorithm. The XTMS scoring function incorporates the thermodynamic favorability of a pathway by both including the sum of all the $\Delta$G values (taken from MetaCyc) of each reaction in a pathway and including the number of unfavorable reactions (any reactions with a $\Delta$G value greater than zero) for each pathway. AGPathFinder [44] uses $\Delta$Gs (in addition to compound similarity) to guide the search as weights.

### Stoichiometry

Graph-based pathfinding methods can incorporate reaction stoichiometry to limit the number of biologically irrelevant pathway results. The carbon flux paths algorithm proposed by Pey et al. [42, 43] introduces steady-state constraints. Pey et al. demonstrate that using carbon flux paths significantly reduces the connectivity of certain compounds, such as oxaloacetate in *E. coli*, compared to a graph-based search without stoichiometric constraints. Introducing stoichiometric constraints allows carbon flux paths to distinguish between oxic and anoxic conditions in *E. coli*, which was not possible in previous graph-based algorithms. However, this pathfinding method was only tested within the metabolic network of a single well-studied organism (*E. coli*) and, like constraint-based methods, is not easily scalable to large multi-organism networks.

### Enzyme efficiency and promiscuity

Enzymes can have different reaction rates, depending on how efficient an enzyme is in converting the substrate to product. On the other hand, promiscuous enzymes can catalyze reactions which may not be found in existing databases and may be used to expand the metabolic pathfinding search. In Cho et al. [54], binding site covalence was factored into ranking enzymes, where the highest ranked enzyme candidates were included in the final pathway solutions. In MRSD [58], edges between compounds are weighted based on the frequency of reactions that use the specified substrate to produce the specified product. This approach does not filter out species duplicates. The XTMS webserver scoring function [12] takes into account a gene score in ranking pathway results found by the RetroPath algorithm. The gene score is calculated for each pathway based on the average of the pathway’s individual reaction scores, which is determined by the estimated promiscuity of the putative enzyme assigned to the given reaction based on the tensor product technique.

## Organism

Many algorithms give the user the ability to select an organism of interest. Arita et al. [22] mention that their search algorithm can find pathways specific to one organism if the user specifies a weighting scheme that heavily penalizes reactions taken from all other organisms. In RouteSearch [40], the user can specify weights for reactions taken from organism vs. reactions taken from a larger library including all organisms. Many others require the user to select which organism or group of organisms to look at [32, 58]. Other methods do not require user input. In Cho et al. [54], enzymes are ranked based on organism specificity. DESHARKY [59] limits the number of compounds that are not organism-specific to only one non-specific reactant and one non-specific product. In GEM-Path [51], there is an association between reactions and organisms. One of the more interesting of these algorithms is MRE [60], where the search takes into account endogenous competition of reactions. By considering which reactions happen more frequently in an organism, pathways can be optimized to include the most common reactions to maximize the production of the target compound and exclude reactions that may only occur at very low rates in the organism.

## Discussion

Pathfinding is a critical and preliminary step in the development of novel biosynthetic pathways. Pathfinding is often done manually, though there are many existing tools that can enumerate putative pathways with minimal input from the user. After a pathway has been identified, much time and effort goes into building, testing, troubleshooting, and optimizing the biological system, and not the initial pathway discovery [61]. This is acknowledged by metabolic engineers and synthetic biologists alike. Assisted pathfinding, for now, is typically restricted to providing and suggesting a series of enzymatic conversions through the aforementioned algorithms and ranking heuristics. It is up to the user to determine what organisms the genes should be sourced from based on limited enzyme kinetic data, which genetic system to use to regulate expression, and which organism to use as an appropriate host. Each step of this process is a challenge, and widespread adoption of assisted pathway discovery algorithms will depend on improved integration with the pathway engineering workflow. For this reason, future directions of assisted pathfinding must include the following: 1) maximizing the utility of existing but limited databases to find paths to non-native or other diverse commodity compounds, 2) facilitating the interpretation of the generated pathway solutions through visualizations and other methods, 3) assisting in gene selection based on known enzyme kinetics and other parameters of enzyme activity, and 4) identifying solutions with specific network topologies such as branched pathways and or cycles.

### Non-native compounds

There has been a recent push to expand searches to non-native compounds using reaction rules, building on BNICE [13, 14], because it is appreciated that the single greatest limiting factor to pathfinding is the completeness of the referenced databases. The ability to find paths to a non-native compound is severely limited when restricted to metabolic databases consisting of almost entirely of native compounds. General reaction rules can substitute for predicted enzyme promiscuity where specific enzyme reactions for a structurally similar but a non-native substrate are needed as either the target or an intermediate in a pathway. Reaction rules can serve as an acceptable best guess or a lead when a pathway cannot be found in its absence. This need has recently lead to the generation of expanded databases (e.g., MINEs [62] and ATLAS [11]) that apply reaction rules to existing databases (e.g., KEGG [63]) to augment them and expand their reach. More work is needed in this area, as our research has identified a number of compounds of interest that still remain outside the reach of these expanded databases.

### Databases

Although the cumulative information that is available across all metabolic databases is extensive, manually searching, gathering, and compiling information from different databases is a challenging task. Each database often has its own representation and set of ID numbers for identifying components like compounds and reactions, in addition to its own organization schema, suited specifically for the intended purposes of the database. These differences make it challenging to determine the exact links and relationships between information in different databases. There have been a few recent efforts to integrate different metabolic databases and create a less redundant, more comprehensive, and more accessible resource for metabolic information (e.g., BKM-react [64], MetRXN [65], and MNXref [66]). The effort to make a more comprehensive, unified metabolic resource could be a great asset to developing new metabolic pathfinding algorithms, as the metabolic representations, heuristics, and constraints used in these algorithms rely heavily on the breadth and completeness of the used metabolic database(s). In addition to this, it would be very helpful for databases to adopt an open distribution model when fiscally reasonable. Restrictions on data distribution hinder further development of pathfinding tools, and licensing barriers make it harder to adopt a single framework.

### Interface and visualization

As the pathfinding capabilities improve, so do the number of solutions that can potentially be generated, and with it the challenge of providing the user with tools to explore the solutions that can number in the thousands and identify pathways of interest. Because of this, there is an increasing amount of user interaction built into pathfinding webservers (see MRSD [58], BioSynther [67], ATLAS [11], and XTMS [12]). By having a more interactive webserver interface, users can quickly modify their queries or filter the results to find the solutions they want. This filtering may be achieved either by ranking as has been previously discussed, clustering of results based on pathway similarity or overlap [68], allowing the user to exclude pathways based on the presence or absence of specific intermediates that the user chooses to avoid, or some mixture of all of these. Improved visualization solutions will provide users with a balance between an abundance of options and ease of identifying promising pathways.

### Gene selection

In addition to visualizations, a well-developed interface could integrate suggestions for genes based on enzyme activity and evidence of heterologous gene expression so that the user can seamlessly transition from pathway discovery to the initial build phase. Databases, such as BRENDA [5], have experimentally determined values for many enzymatic characteristics that could be used in determining the gene of choice for each reaction step. However, this information has yet to be implemented in a pathway discovery and selection webserver.

### Topology

Almost all pathfinding algorithms are limited to producing linear pathways with a few exceptions [41, 69]. Branched pathways and cycles represent different topologies of metabolic networks that are of interest to metabolic engineering because the resulting condensation or recycling of constituent material can potentially improve the theoretical yield for a pathway. Though linear pathways are sufficient in most cases, the capability of identifying more complex and efficient pathways would be desirable.

### Conclusion

Ultimately, the best pathfinding algorithm is the one that suits the user’s needs and is paired with an interface that facilitates pathway discovery. Pathfinding webservers can assist with the design of novel, feasible, and hopefully improved pathways, but as discussed, pathfinding needs to become more highly integrated with the entire process of metabolic engineering. This survey of the available features and future directions aims to increase adoption of existing pathfinding tools while advocating for advancements that will increase their utility.
